# Estrogen lowers triglyceride via regulating hepatic APOA5 expression

**DOI:** 10.1186/s12944-017-0463-0

**Published:** 2017-04-04

**Authors:** Fei Luo, Yuan Guo, Gui-yun Ruan, Ran Peng, Xiang-ping Li

**Affiliations:** Department of Cardiovascular Medicine, The Second Xiangya Hospital, Central South University, 139 Middle Renmin Road, Changsha, Hunan 410011 People’s Republic of China

**Keywords:** Estrogen, Triglyceride, GPR30, APOA5

## Abstract

Estrogen had been found to be negatively associated with serum triglyceride (TG) levels. Apolipoprotein A5 (APOA5), a novel member of apolipoprotein family, was reported to have a strong ability to decrease serum concentrations of TG. Clinical data found concentrations of APOA5 were higher in woman than that in men, and the negative relationship between APOA5 and TG levels was more significant in woman. These suggests APOA5 may involve in estrogen actions. Therefore, we hypothesize estrogen up-regulates serum concentrations of APOA5 and subsequently decreases serum TG levels. We will design the following experiments to test this hypothesis. (1) We will treat wild and APOA5-defeted ovariectomized hamster with or without estrogen to examine if estrogen could up-regulate concentrations of APOA5 and decrease TG levels. (2) We will treat HepG2 cells with estrogen and investigate the possible mechanisms.

## Background

Estrogen, an essential female hormone, is critical to modulating lipid metabolism [1]. Decline in estrogen concentrations was linked to dyslipidemia in peri- and post-menopausal women, including increased serum triglyceride (TG) levels [2, 3]. Guo et al. [2] reported that TG levels of postmenopausal women were significant higher than that of pre-menopausal women (1.73 ± 0.98 vs 1.07 ± 0.63 mmol/L, *P* < 0.05). Whitcroft et al. [4] reported that transdermal estradiol treatment for post-menopausal women could lower serum TG levels by about 16.4% (*P* < 0.01). Pulchinelli et al. [5] found that estrogen replacement therapy statistically reduced TG levels of hysterectomized women. A meta-analyzed further confirmed a modest effect of transdermal estradiol treatment on decrease of serum TG levels [6]. Recently, estrogen was confirmed to reduce TG levels in in mice hepatology [7]. Therefore, estrogen is important in maintaining TG homeostasis, but the underlying mechanisms remain to be elucidated.

Apolipoprotein A5 (APOA5) was a novel member of apolipoprotein family and mainly secreted by liver, which possessed a strong ability of decreasing serum TG levels [8]. *APOA5* genetic variants were associated with increased TG levels in humans, and human *APOA5* transgenic mice showed a decrease in plasma triglyceride concentrations to one-third of those in control mice [9–12]. Moreover, data was not only reported a negative correlation between APOA5 and TG levels, but also indicated the two indices changing more significant in female. Zhao et al. [13] reported serum concentrations of APOA5 were negatively and predominantly correlated with TG levels in female (*r* = −0.496, *P* = 0.001) but not in male (*r* = 0.054, *P* = 0.709). Ishihara et al. [14] observed serum concentrations of APOA5 in premenopausal female were higher than that in male of the similar age. It suggests estrogen might up-regulate APOA5 expression, and thereby decrease TG levels.

## Presentation of the hypothesis

The estrogen actions had been traditionally attributed to the classical nuclear estrogen receptors (ERs), ERα and ERβ. More recently, the G protein-coupled receptor 30 (GPR30), also known as G protein-coupled estrogen receptor 1, was claimed as an essential estrogen receptor [15]. It was also identified to be closely related to lipid metabolism. The *Gpr30* knockout mice exhibited an abnormal lipid profile with an approximately 71% increase in triglyceride levels [16, 17]. Consistently, Zucchetti et al. [18] demonstrated that GPR30 was essential for estrogen exerting its function in liver of altering canalicular transporter function and localization. This indicates that estrogen regulates TG at least in part through GPR30.

Peroxisome proliferator-activated receptor α (PPARα) was a number of the nuclear receptor superfamily and directly regulated lipid transport, storage and metabolism. PPARα was also found to up-regulate *APOA5* gene expression and a functional PPARα response element in the proximal *APOA5* promoter was detected by using deletion and mutagenesis analyses [19]. Furthermore, it was identified that hepatocyte nuclear factor-4α (HNF-4α) was a highly conserved member of the nuclear receptor superfamily, which was initially identified as a transcriptional factor required for liver-specific gene expression, and it was also critical in regulating the transcription of genes involved in glucose and lipid metabolism including *APOA5* [20]. Intriguingly, previous study reported that HNF-4α and PPARα expression could be activated by hepatic protein kinase A (PKA) pathway [21]. More interestingly, it was reported that hepatic GPR30 combined with estrogen and in turn exerted its function by activating PKA pathway [18]. Thus, we speculated estrogen combined with GPR30 and consequently activated the hepatocyte PKA signaling pathway, which enhanced PPARα and HNF4α expression in liver and thereby increasing hepatic APOA5 expression and finally decreased serum TG levels.

Therefore, we hypothesize estrogen up-regulates APOA5 expression to reduce plasma TG levels via combination with GPR30 with an aim of providing more evidence for exploring the TG lowing effect of estrogen and insight into novel therapeutic target.

## Testing the hypothesis

We will design some experiments to test this hypothesis. (1) We will treat wild ovariectomized hamster with or without estradiol to examine if estradiol could up-regulate APOA5 and decrease TG levels. Then we will evaluate whether the deletion of APOA5 could abort the decrease effects of estradiol. (2) We will treat HepG2 cells with estradiol and detect the concentrations of APOA5 in and out cells. We will also use GPR30 receptor antagonist to exam if this effect was induced by GPR30.

## Abbreviations

APOA5: Apolipoprotein A5
ERs: Estrogen receptors
GPR30: G protein-coupled receptor 30
HNF-4α: Hepatocyte nuclear factor-4α
PKA: Protein kinase A
PPARα: Peroxisome proliferator-activated receptor α
TG: Triglyceride
